# Sensorless Speed Estimation for the Diagnosis of Induction Motors via MCSA. Review and Commercial Devices Analysis †

**DOI:** 10.3390/s21155037

**Published:** 2021-07-25

**Authors:** Jorge Bonet-Jara, Alfredo Quijano-Lopez, Daniel Morinigo-Sotelo, Joan Pons-Llinares

**Affiliations:** 1 Instituto Tecnológico de la Energía, Universitat Politècnica de València, Camino de Vera s/n, 46022 Valencia, Spain; jorboja@die.upv.es (J.B.-J.); aquijano@ite.upv.es (A.Q.-L.); 2 Research Group ADIRE, Institute of Advanced Production Technologies (ITAP), University of Valladolid, 47011 Valladolid, Spain; daniel.morinigo@eii.uva.es

**Keywords:** fault diagnosis, induction motors, MCSA, sensorless speed estimation, Industry 4.0

## Abstract

Sensorless speed estimation has been extensively studied for its use in control schemes. Nevertheless, it is also a key step when applying Motor Current Signature Analysis to induction motor diagnosis: accurate speed estimation is vital to locate fault harmonics, and prevent false positives and false negatives, as shown at the beginning of the paper through a real industrial case. Unfortunately, existing sensorless speed estimation techniques either do not provide enough precision for this purpose or have limited applicability. Currently, this is preventing Industry 4.0 from having a precise and automatic system to monitor the motor condition. Despite its importance, there is no research published reviewing this topic. To fill this gap, this paper investigates, from both theoretical background and an industrial application perspective, the reasons behind these problems. Therefore, the families of sensorless speed estimation techniques, mainly conceived for sensorless control, are here reviewed and thoroughly analyzed from the perspective of their use for diagnosis. Moreover, the algorithms implemented in the two leading commercial diagnostic devices are analyzed using real examples from a database of industrial measurements belonging to 79 induction motors. The analysis and discussion through the paper are synthesized to summarize the lacks and weaknesses of the industry application of these methods, which helps to highlight the open problems, challenges and research prospects, showing the direction in which research efforts have to be made to solve this important problem.

## 1. Introduction

Over the last three decades, Sensorless Speed Estimation (SSE) for rotating machines has undergone a great advance. From the very first moment, SSE techniques were mainly applied in the field of electric motor control (e.g., Field Oriented Control) [1]. Therefore, their development has always been linked to the improvement of control methods and frequency converters. However, despite their natural link to sensorless control, SSE algorithms also play a key role in fault detection methods for Induction Machines (IMs), specifically in those that assess motor condition by localizing speed-dependent fault harmonics in the frequency spectrum of the stator current [2,3,4,5,6]. First, because in order to extract and quantify these harmonics, a good speed estimation is previously needed to accurately determine their position [7,8]. Second, because most industrial motors are neither coupled to physical speed sensors nor do they have the shaft accessible to perform manual measurements. Yet, although the basis is the same, the requirements that SSE methods have to meet depend on the field of application.

On the one hand, in order to carry out a good control, it is imperative to obtain a real-time response of the estimated parameters, specifically, that of the speed. This necessary real-time response generally involves a trade-off with accuracy, as it requires the SSE algorithm to use short voltage and current records. Furthermore, speed estimation methods also have to meet the requirement of a stable performance over a wide range to provide more versatility (e.g., low-speed operation). Regarding these two needs, in the last decade, an effort has been made to increase accuracy and stability using modern techniques such as neural networks, genetic algorithms, etc. [9,10,11,12,13,14,15,16,17]. Nevertheless, since the possibility of working with large data records is restricted in the field of controlled AC drives, the maximum degree of accuracy is yet to be fully exploited.

On the other hand, there are procedures in the field of IM diagnosis in which SSE algorithms can be implemented, where time response and data record length are less critical. As a consequence, accuracy can be increased. One of these procedures is Motor Current Signature Analysis (MCSA). This method is mainly applied to detect speed-dependent fault harmonics in the frequency spectrum of stator current [18], although other magnitudes can also be used (e.g., instantaneous power, reactive power or apparent power [19,20,21]). Many research papers, whose main purpose is not localizing the fault harmonic, since they test lab motors with perfectly-known conditions, analyze the expected fault harmonic frequency band assuming that the highest peak of the band will be the fault harmonic [22,23,24,25,26,27,28,29,30]. Some other authors use filters as wavelet transform to extract sub-signals related to frequency bands where the harmonic is supposed to be [31,32,33]. Nevertheless, these are dangerous strategies in real non controlled industrial environments, since these harmonics can show frequencies very close to those of healthy state caused by multiple factors (winding harmonics, load oscillations, gear boxes, etc.), and be confused with them. To avoid these false positives and negatives, some authors directly measure the speed to localize the faulty harmonics perfectly [34,35]. Nevertheless, speed sensors are rarely present in industrial environments. Therefore, a SSE algorithm with high accuracy is vital to precisely track fault harmonics and reduce both false positives and negatives. In this regard, SSE techniques based on extracting speed-dependent harmonics (fault or healthy) using the Fast Fourier Transform (FFT) can increase the degree of precision without using sophisticated signal processing methods. This might be done by recording long periods of stator current (50 s, 100 s, even 200 s) in applications with little load oscillations and connected to stable grids (as is the case for a large number of industrial applications), which in turn would not be a problem for the diagnostic method, since rotor bar and eccentricity faults, usually detected by MCSA evolve slowly. Moreover, as both the diagnostic method and the SSE method would use the FFT to extract respectively fault and healthy/fault harmonics of the same stator current, they could be put together in one single algorithm. Finally, other authors have proposed to measure additional quantities as the external flux [36] to estimate the speed; nevertheless, this leads to a less robust method, as the flux sensor must be placed near the motor housing, which in some applications is not possible (e.g., submersible motor-pumps).

SSE techniques, regardless of whether they are used for diagnostic or sensorless control, can be classified into two large groups of methods: Fundamental Model-Based (FMB) [1,9,10,11,12,13,14,15,16,17,37,38,39,40,41,42,43,44,45,46,47,48,49,50,51,52,53,54,55,56,57,58] and Magnetic Anisotropy-Based (MAB). The latter can be subdivided into two more groups of methods: Signal Injection-Based (SIB) [59,60,61,62,63,64] and Slotting and Eccentricity Harmonics Based (SaEHB) [4,65,66,67,68,69,70,71,72,73,74,75,76,77,78,79]:

- FMB methods require to estimate or to know in advance a wide range of parameters (stator resistance, inductance, rotor time constant, number of slots, etc.). As many of them are time-varying (e.g., stator resistance can have variations in a range of 1:2 [60]), there are two possible scenarios: if they are assumed to be constant, an error is added to the speed estimation; on the other hand, if they are estimated over time, the algorithm can get more complicated and unstable.
- SIB methods do no depend on any time-varying parameter. However, they require a much more complicated implementation, and besides, not all drives are compatible with their requirements [61,80]. All this makes them hardly applicable on an industrial scale.
- SaEHB techniques do not depend on any time-varying parameter either. Yet, when based on Rotor Slot Harmonics, they depend on a machine characteristic that is rarely known by the motor owner: the number of rotor slots. Conversely, if they are based on Mixed Eccentricity Harmonics, which only depend on an easy to know parameter (number of poles), precision and detectability problems may arise due to their narrow bandwidth [72].

The previous knowledge of specific parameters or the need to estimate them is a classic problem in SSE techniques no matter if they are used in diagnostic or control applications. This means a big drawback when it comes to obtaining a general method that can be applied to any motor in industry. That is because, in most cases, operators and technicians do not know data beyond those indicated on the nameplate.

Therefore, online condition assessment methods based on frequency spectrum information need a precise SSE algorithm that allows to automatically localize speed-dependent fault harmonics and reduce both false positives and negatives. Moreover, the set formed by the SSE algorithm and the diagnostic method should also be able to be easily integrated into Industry 4.0 systems in order to provide a complete monitoring of motor condition over time. Traditional SSE control-oriented methods are not a good option for these purposes as they provide a real-time response at the expense of a lower accuracy. Yet, if integrated into diagnostic procedures, and thus relieved of a need for real-time response, some of the traditional SSE techniques might be able to provide a much more precise fault harmonic positioning, which in turn would translate into a much more reliable diagnostic.

The paper analyses for the first time the SSE methods present in the technical literature from the perspective of its application to induction motors diagnosis via MCSA. This work aims to reveal which are the open problems, challenges and research prospects that the scientific community has yet to work on to finally bring a precise, general and automatic algorithm able to work in the context of a 4.0 industry. To that end, the limitations of each technique are analyzed, from both theoretical and industry applications perspective. First, taking as a starting point the work presented at [81], this paper shows, through an industrial case (Section 2), how important an accurate and automatic SSE is for fault diagnostic methods based on localizing, extracting and quantifying speed-dependent harmonics. Then, the paper thoroughly reviews and analyzes the two main groups of SSE techniques, pointing out their limitations when applied with diagnostic procedures (Section 3 and Section 4). Moreover, using again industrial cases thanks to a database of industrial measurements belonging to 79 IM, it is shown how the two leading commercial devices for diagnosis work, which are the principles of the algorithms they implement and what are their weakness and sources of errors (Section 5). Concluding, SSE in diagnostic procedures is not a solved issue; thus, the analysis and discussion performed in this paper, related to the industry application of these techniques, enables to finally synthesize and highlight the lines of research in which academia should focus its efforts in order to provide a truly effective SSE industrial method that could help obtain automatic and reliable diagnostic results.

## 2. Importance of SSE in Diagnosis

Speed estimation is a key factor in steady-state fault diagnosis via MCSA. Certain faults, such as rotor bars breakage and eccentricities, induce speed-dependent harmonics in the stator current. For example, the frequencies of the main harmonics related to rotor bars breakage in the spectrum of the stator current are given by:

(1) $$f_{BBH}=\left(1\pm 2ks\right)f_{0}$$

When *k* is equal to one, those harmonics are commonly known as the Upper Sideband Harmonic (USH), positive sign, and the Lower Sideband Harmonic (LSH), negative sign. The fundamental frequency $\left(f_{0}\right)$ can be easily identified as it is the highest peak in the spectrum. Then, if the speed is also known or estimated, the slip (s) can be determined and with it the position of these harmonics. Finally, they can be quantified in order to issue a diagnosis.

Regarding the diagnosis, from now on, the following criterion will be used to determine the condition of the rotor bars in an IM: if the amplitude is below −48 dB the IM is considered to be healthy, between −48 and −36 dB there might be one or several broken/cracked bars, and above −36, there are multiple broken bars. This criterion is well established in the field of diagnosis [7], proved valid for different sizes of motors [18,82] and shared by commercial devices (with slight variations in the thresholds, as shown in Section 5). It is also worth mentioning that, as these are empirically obtained limits, two different motors with the same amplitude of broken bar harmonics may have different numbers of broken or cracked bars. Yet, it should not be forgotten that the aim of the examples that will be used in this paper is to show that: given a failure criterion, a small error in speed estimation can lead to a wrong diagnosis. Therefore, the important point here analyzed is if the speed estimation method allows to precisely determine the frequency of the fault harmonic, while the exact value of the fault thresholds is secondary.

**Figure 1:**
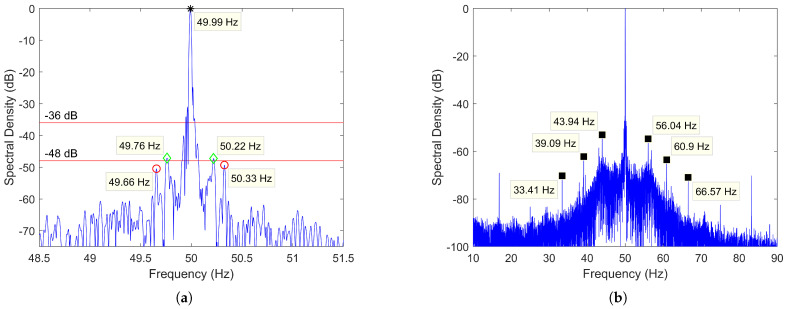
(**a**) Stator current spectrum of a two-pole 90 kW IM with healthy state harmonics (diamonds) close to the broken bar harmonics (circles) and (**b**) zoomed out spectrum showing the smearing effect around the fundamental and other harmonics caused by load oscillations.

Next, to illustrate how important it is to know the speed accurately, the current of a two-pole 90 kW IM driving a submerged pump has been analyzed by the FFT. Figure 1a shows the spectrum of the stator current (blue line) with the fault thresholds (red lines), in which a deviation of only 3 rpm means to identify two healthy state harmonics (marked with green diamonds) as two broken bar harmonics (marked with red circles). The harmonics marked with green diamonds belong to the healthy state since they do not satisfy any of the formulas related to rotor electrical asymmetries, rotor-stator misalignments and stator electrical asymmetries. They could be caused by the operating conditions of the load, since after zooming out the spectrum (Figure 1b), it can be observed a smearing effect around the fundamental frequency, as well as several sets of sideband harmonics not predicted by (1), but separated from the fundamental a multiple of a certain bandwidth: as stated in [18], these effects are usually related to the operation with gear boxes and/or load oscillations. Therefore, the error leads to diagnose the motor as faulty (green diamonds within the red lines) when, actually, the broken bars harmonics are in the healthy zone (red circles below red lines).

From the above example, it is clear the need for accurate speed information in order to make a reliable diagnosis given a certain fault threshold. This information can come from a physical speed sensor or be estimated using an SSE technique. The cost of a physical sensor increases proportionally to the level of accuracy required, being sometimes a significant amount of the total price of the IM, especially when a custom design is required for its correct coupling. Moreover, they are very sensitive to the conditions of operation and location. Variations in temperature or large lengths of data transmission cable can lead to erroneous measurements. In addition, a precise and careful assembly on the shaft is required. Conversely, sensorless techniques are able to overcome these drawbacks since they are: lower-cost options for the same accuracy (easy integration in existing control and measuring devices), more robust (measurements far from motor location implies better isolation; it also prevents motor disassembling in case of problems in measurement systems), and the only reliable option in certain industrial applications where physical sensors are very difficult to install (e.g., pumps submerged at great depths).

Alternatively, some authors propose to avoid the speed estimation problem by diagnosing the IM based on the following principle: since the fault-related harmonics have a certain operating bandwidth, determined for instance by (1) varying the slip from 0 to its rated value, it is possible to locate the fault harmonics in the spectrum by calculating the maximum in that bandwidth after a pre-treatment process [31,83]. This diagnostic procedure relies on the fact that no other significant harmonic will be present in the search band. Nevertheless, as seen through the example presented in this section, that does not always hold true, and therefore, it can be a source of false positives. Thus, an algorithm that can accurately estimate speed, and consequently, the position of fault harmonics, is essential in the predictive maintenance of IM via MCSA.

Furthermore, in recent years, new requirements have started to emerge among those industries that are adopting an Industry 4.0 philosophy: collect considerable amounts of data related to different systems, process them with specific algorithms to obtain information and act through this set of interconnected systems to achieve optimal autonomous production. In terms of diagnosis, this means:

- Real-time condition monitoring: motors must be continuously diagnosed to achieve perfect operation.
- Noninvasive methods: production must not be altered (just when maintenance is needed).
- High reliability: avoid actions based on a false diagnosis.
- Automated process without human intervention: efficient and fast diagnosis of big amounts of motors.
- Intelligent operation of the facility: take the proper actions based on a correct diagnosis.
- Interoperability via the Internet of Things: information related to the state of the machines must be shared with the rest of the systems and take decisions collectively.

Then, there is not only the need for reliable and accurate diagnostic algorithms, but also for them to operate continuously, fully autonomously without requiring an expert intervention, and without altering the production process. Nevertheless, as it will be demonstrated later, current SSE algorithms implemented in commercial diagnostic devices do not meet these requirements, as they need human intervention to double-check the diagnostic due to the lack of reliability. The reliability is further reduced when the motor works in the presence of load oscillations and is fed by a frequency converter, the latter condition becoming more and more common in certain industries (e.g., water supply facilities). Moreover, it will also be shown that even those techniques that are able to provide a very accurate speed estimation and to be compatible with MCSA, also need the intervention of an expert to determine some of their initial parameters. Therefore, academia has yet to provide the modern industry with high-precision and automatic SSE techniques that can serve as the cornerstone for the development of more reliable continuous condition monitoring systems based on MCSA.

## 3. Methods Based on the Fundamental Model

In this section, FMB methods are described, reviewing both the two most common techniques (Section 3.1 and Section 3.2), and their respective improvements through the use of Artificial Intelligence (AI) (Section 3.3). Nevertheless, the key point, once the methods are reviewed, is to analyze their suitability for their use in IM diagnosis via MCSA (Section 3.4).

**Figure 2:**
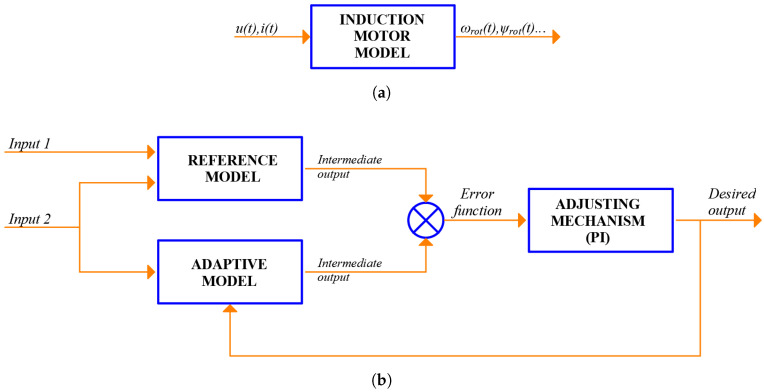
(**a**) Open-loop model and (**b**) a traditional scheme for a closed-loop MRAS.

FMB methods describe the machine assuming a sinusoidal distribution of the air-gap flux (spatial harmonics of a higher order than the fundamental are neglected) and mainly using the d-q axes as the reference system. An open-loop scheme, depicted in Figure 2a, is their simple way of implementation: the model outputs the speed (among other variables) using as inputs voltages and currents. However, this open loop model does not take into account the variations in parameters that occur during normal machine operation, such as the change in stator resistance with temperature [84]. Hence, as the model parameters remain constant but not the machine ones, there are deviations between the estimated speed and the actual one. This problem can be mitigated using closed-loop schemes (Figure 2b) where error signals between measured and estimated magnitudes are used to adapt the response and/or the parameters of the model. Despite the variety of closed-loop schemes that exist, they can be subdivided into: Model Reference Adaptive Systems (MRASs), observer schemes and schemes that incorporate AI.

Within the second group, there are different types of observers such as Sliding Mode Observer [48], Luenberger Observer [49], or observers based on the Extended Kalman Filter (EKF) [50,51,52,53,54,55,56,57]. Although with different characteristics from the control point of view (SMO and LO have better parameter robustness, dynamic performance and low-speed operation, while EKF has the best noise immunity [58]), their advantages and disadvantages regarding their implementation in online diagnosis via MCSA are very similar (pros and cons later analyzed). Moreover, in high-speed steady-state operation, which is the range where MCSA is applied, the three observers exhibit very similar accuracy (relative error of 0.2% at 1500 rpm: perform similarly in the MCSA operation zone). Therefore, since the aim of this paper is not to review in detail each of the observer schemes, but to present their general characteristics and then analyze their compatibility with MCSA, only the EKF will be used as an example. The reasons behind this selection is that this type of scheme is one of the most used in the problem of speed estimation in the induction motor, thanks to its ability to deal with model uncertainties, measurement noise and its nonlinearity.

### 3.1. Model Reference Adaptive Systems

MRASs are schemes that consist of a reference model, an adaptive model and an adjustment mechanism (Figure 2b). Reference and adaptive models estimate the same intermediate output but from different inputs. The desired magnitude to be estimated, in this case speed, is an input of the adaptive model, but not of the reference model. Finally, speed is estimated by minimizing the error between the intermediate outputs through a controller.

The first MRAS for SSE used the rotor flux as the error function and was presented by Tamai et al. [37] in 1985. However, it was complex, highly sensitive to machine parameter variations and unstable under certain conditions. On the one hand, complexity was mainly addressed in [38] (1992, Schauder) through a simplified and modified version of Tamai’s work that rapidly became popular for its ease of implementation. On the other hand, sensitivity to parameter variations and instability were addressed through the use of other error functions such as back-EMF [39], reactive power [40], stator current [41] or fictional quantities [42]. Two excellent and wider reviews on these different MRAS schemes were conducted in [1,43]. From these works, it can be concluded that classical reactive power-error-based MRAS schemes are the best to provide insensitivity to stator resistance variations, while classical fictional quantities-error-based MRAS schemes are a better option to provide stability in all zones of operation. In this regard, providing MRAS schemes that combine both qualities has been an aim of academia in recent years [44,45,46]. Finally, other authors have focused their research in reducing the computational time by replacing the PI controller with other structures [47].

### 3.2. Extended Kalman Filter Observer

An observer scheme is a dynamic structure that uses a model of the real system (e.g., IM) to estimate internal/no-measurable variables (e.g., flux, speed, position, etc.) from measurable inputs/outputs (e.g., stator currents and voltages). In the case of the EKF, the machine is modeled as a non-linear 5th-order system, where the mechanical speed is regarded as an additional state variable. Using this system, the EKF applies a two-stage recursive algorithm with a stochastic approach that accounts for the noise in the system, regarded as a Gaussian white noise environment. Then, in the first stage of the algorithm, the state variables are predicted, while in the second stage (filtering stage), the predicted variables are corrected.

One of the first successful applications of the EKF observer for SSE in IM was presented by Kim et al. in 1994 [50]. Since then, different approaches have been presented to increase speed accuracy. In [51], the problem was addressed from the perspective of improving the correct choice of noise covariance and weight matrices, while in [52] from the perspective of simultaneous estimation of speed and rotor resistance variations. Finally, in [53], the approach was based on using an observer that does not use any linearization: the UKF. Using this observer, the authors stated that it was possible to obtain better results than using the EKF, especially in low-cost applications. However, this claim has been recently disputed in [54]. In this study, a comprehensive comparison of both methods is done under challenging conditions. It is shown that while equal in terms of performance, EKF is superior in terms of computational burdens and therefore a better choice for IM estimation problems. Yet, it is still debated which may be the best observer for the IM speed estimation problem. In this regard, works continue to be published where modified versions of the EKF are proposed to deal with the classical problems of choosing the noise covariance matrices and increasing the robustness against parameter variations [55,56,57].

### 3.3. Artificial Intelligence

In the early 2000s, AI techniques emerged as complementary tools for MRAS and EKF closed-loop schemes aimed to overcome three of their major problems: complexity of non-linear mathematical models, instability and parameter compensation [80]. Among them, the most used for these purposes are: Artificial Neural Networks [9,10,12] (ANNs), Fuzzy Logic [13,15] (FL) and Genetic Algorithms [16,17] (GAs).

Having the role of complementary tools, these techniques are used to replace parts of closed loop schemes in order to obtain a better performance in the estimation process. For example, in [9], an ANN system replaced the adjustment mechanism of a classic MRAS scheme for a more robust behavior against machine parameter variations, while in [10], another ANN system replaced the adaptive mathematical model to improve stability in the four quadrants of operation. In [11], the approach was to use two kinds of ANN simultaneously: one for rotor speed estimation and the other for rotor resistance. In [12], different training algorithms for an ANN that replaces the flux estimator of a MRAS scheme were compared. In [13], a FL system replaced the adjustment mechanism of an MRAS scheme in order to obtain a synchronous speed estimator unaffected by variations in temperature. In [14], an adaptive supervisory sliding fuzzy cerebellar model is used as a speed controller in order to maintain the speed error within predefined boundaries. In [15], an FL control mechanism was used for stator resistance adaptation in order to improve speed estimation. In [16,17], GAs were used to optimize the right choice of covariance filters matrices so as to enhance dynamic performance of EKF speed estimators. However, although these techniques have been proved to be a good tool to minimize the effects of parameter variations, their larger computational time and their need for training (ANN) can be a drawback in industrial applications.

### 3.4. Methods Based on the Fundamental Model in Online Diagnosis

Since the early nineties, all efforts have been put into developing FMB speed estimation techniques for sensorless control. No specific research has been done on developing these methods for diagnostic procedures. This may be due to the fact that the requirements of MCSA and FMB techniques do not fit together easily in an industrial environment, especially when implemented in portable devices, as explained below.

On the one hand, an MCSA-based commercial portable device needs to be as less invasive as possible: the diagnostic must be performed online and without interfering with the production process. MCSA only needs to sense stator currents (sometimes just one), whereas FMB methods need to sense both stator currents and voltages (inputs of the reference or adaptive models). In this regard, although current sensing can be done in a non-invasive way using current clamps, voltage sensing requires the motor to be stopped in order to couple the probes without electrical hazard. Furthermore, another inconvenient from an industrial point of view is that in order to carry out the first stage of parameter identification that these methods require (parameters of the model), it is necessary to purposely stop the motor or wait for a scheduled stop.

On the other hand, a MCSA diagnostic procedure also needs to be as reliable as possible. Reliability is greatly improved if an accurate speed estimation algorithm is used. In FMB methods, the degree of accuracy depends on the degree of robustness against parameter variations. Over the last few years, there has been a considerably improvement in this regard. Current methods, such as [46,57], can now obtain maximum errors of only a few rpm when there are changes in the parameters. However, while this degree of accuracy may be enough for sensorless control, this might not hold true for MCSA, especially at low slips and in an industrial environment. As shown in Section 2, only an error of 3 rpm is enough to issue a false positive. Moreover, if the motor is also working with load oscillations, the error needed to commit a mistake is even lower. Therefore, it is always preferable to use an SSE technique that does not depend on any changing parameter.

All the aforementioned drawbacks make FMB methods seemingly unsuitable for providing the speed information required by MCSA-based portable devices. However, MCSA-based continuous monitoring systems can open the door to use FMB techniques. If implemented in the driver, the set formed by the MCSA diagnostic procedure and the FMB method could use the same voltage and current measurements as the control system. Moreover, the system could also take advantage of the natural stops to perform the parameter identification. If designed as a separate device, it could be installed in the distribution board, and the same would apply. Yet, it is still necessary to study whether the accuracy provided by a method like this is enough for its use in high-reliability diagnostic procedures via MCSA in comparison with other SSE techniques. Furthermore, if complemented with AI, it would also be necessary to address in future comparative research, their feasibility in diagnostic devices, as these techniques require previous training and are computationally intensive.

## 4. Methods Based on Magnetic Anisotropy

These techniques exploit the effects of magnetic saturation and rotor slotting in IM. Their main advantage is their total independence to machine parameter variations (stator resistance, rotor time constant, etc.). This makes them really robust and often preferable to FMB methods. However, the existence or lack of certain anisotropies, as well as the intensity with which the effects due to slotting are manifested, depend on the machine design, which may reduce the applicability. Therefore, in many cases, a preliminary study of the motor (number of bars, rotor type, depth of slots, etc.) is required.

MAB methods can be classified into two groups: Signal Injection Based methods and Slotting and Eccentricity Harmonics Based methods. In Section 4.1, only a general description of SIB methods is given (without going into reviewing its evolution through time), since these techniques were developed to meet a specific need for control systems that is not found in diagnosis via MCSA. For this purpose, three excellent papers published by Holtz [59,60,61] are used. Conversely, in Section 4.2, the description and review of SaEHB methods are more in-depth and extensive, given that these techniques are more suitable to be used in diagnostic procedures in an Industry 4.0 environment, as analyzed in Section 4.3.

### 4.1. Methods Based on Signal Injection

SIB methods were born in the late 1990s as a response to the unstable performance of FMB at low or zero stator frequency. At these frequencies, the voltage induced in stator by rotor currents is practically zero, which makes the model unobservable: it is not possible to obtain information on rotor dynamics from stator terminals. However, it is possible to obtain accurate information about rotor positon and rotor speed if the machine magnetic anisotropies are exploited [59].

Magnetic anisotropies can be caused in an IM mainly by: magnetic saturation of the fundamental field and the discrete structure of the squirrel cage. However, as Holtz states in [60], “a rotor may be custom designed so as to exhibit periodic variations within a fundamental pole pitch of local magnetic or electrical characteristics”. An example of this could be the periodic variation of the widths of rotor slot openings, the resistance of the outer conductors or the depths of the rotors bars [60].

The technique consists of adding a high frequency and low amplitude voltage to the fundamental excitation in order to exploit these anisotropies. As explained by Holtz in [61]: “The resulting high-frequency currents generate flux linkages that close through the leakage paths in the stator and the rotor, leaving the mutual flux linkage with the fundamental wave almost unaffected”. This signal, called carrier, is modulated periodically by the spatial orientation of the magnetic anisotropies present in the IM. Finally, by extracting and processing the modulated carrier it is possible to obtain information about the rotor angle and the rotor speed [61].

The injected signal can be of two types: a revolving signal [62] or an alternating signal [63]. On the one hand, revolving carriers provide a general view of all machine positions in order to locate the spatial orientation of a particular machine anisotropy. On the other hand, alternating carriers provide a high-sensitive view in a specific spatial direction, which is chosen based on previous knowledge [61]. As an alternative to revolving or alternating carriers, transient signals injected by PWM switches can be exploited. In words of Holtz in [61]: “the transient flux components, owing to their high-frequency content, do not penetrate sufficiently fast through rotor surface to establish mutual flux linkages. These fluxes, instead, create only separate linkages with the respective stator and rotor windings, thus contributing to the total leakage flux”. Therefore, as the mutual flux also remains unaffected, the same principles apply as when external signals are used. Finally, it is also possible to use AI methods in order to improve some of the drawbacks of the technique, such as the need of having to determine, with the help of an expert, the magnetic fingerprint of each machine [64].

### 4.2. Methods Based on Slotting and Eccentricity Harmonics

Unlike SIB methods, SaEHB algorithms exploit magnetic anisotropies using the machine response to the fundamental excitation signal and its low-order harmonics $\left(3\mathrm{rd},5\mathrm{th},7\mathrm{th}\ldots \right)$. Particularly, these techniques track those anisotropies that are due to slotting and constructional/coupling imbalances such as static or dynamic eccentricity. In a simplified and brief way, the discrete nature of the squirrel cage bars causes the rotor system to generate MMF spatial harmonics as well as a periodic variation of the air-gap permeance. By interacting, they produce air-gap flux components that are responsible for inducing a set of harmonics in the stator windings called Rotor Slot Harmonics (RSH). When there are also misalignments between the rotor and the stator, the air-gap permeance is further modified, giving rise to additional air-gap flux components that can induce, depending on the characteristic of the misalignment, Static Eccentricity Harmonics (SEH), Dynamic Eccentricity Harmonics (DEH) or Mixed Eccentricity Harmonics (MEH). The relationship between the frequencies of these harmonics in the current stator spectrum and the machine characteristics has been extensively studied in [85,86] and can be given in a compact form by:

(2) $$f_{h}=\left[\frac{\left(kR\pm n_{d}\right)}{p}\left(1-s\right)\pm \nu \right]f_{0}$$

(3) $$f_{MEH}=\left[1\pm k\frac{\left(1-s\right)}{p}\right]f_{0}$$

where *k* is a natural number 1,2,3…, *p* the number of fundamental pole pairs, *s* the slip, ν the order of the stator time harmonic present in the power supply 1,3,5…, $f_{0}$ the fundamental excitation frequency, *R* the number of rotor bars, $n_{d}=0$ for both SEH and RSH and $n_{d}=1,2,3\ldots$ for DEH. Finally, when *k* is equal to one in (3), those harmonics are known as the Upper Mixed Eccentricity Harmonic (UMEH), positive sign, and the Lower Mixed Eccentricity Harmonic (LMEH), negative sign.

As can be seen, both equations contain information on motor speed through the slip. Therefore, what these techniques do is to process the line current in order to determine the frequency of one or more of these harmonics and then, using (2) or (3), calculate the slip and with it, the mechanical speed. Finally, among the four sets of harmonics, RSH and MEH are usually the targets of these algorithms, since they are present in most IM.

The first works where RSH and MEH were used can be traced back to the middle of the 1980s and the beginning of the 1990s. In 1984, Ishida et al. [65] presented a work where, knowing the number of rotor bars and using analogue filters and zero cross detection, they were able to extract the RSH. This method achieved an accuracy of 0.1 Hz and a minimum operating frequency of 10 Hz. In a later work (Williams et al., 1990, [66]), MEH together with switched-capacitor filters and phase lock loops were used to estimate the rotor speed. In this case, no information about precision was provided. These methods laid down the foundations for the use of RSH and MEH in SSE.

During the 1990s, the accuracy and operating range of these SSE techniques was improved thanks to the use of digital methods such as FFT. Regarding this, RSH were first extracted using FFT in steady-state operation and over a wide range of load levels by A. Ferrah et al., in 1992 [67]. Results showed errors between −10 and 10 rpm when compared to an opto-electrical speed transducer. Furthermore, the speed detector performed satisfactorily down to 2 Hz and had a time response of 3 s. On the other hand, since the harmonics being tracked were RSH, the number of rotor slots needed to be known. Trying to address the problem, they presented a method to determine this parameter from a set of stator current records at rated frequency and under decreasing load levels. In 1996, a new improved method with a maximum error of 5 rpm, a time response of 1 s and an operating range down to 1 Hz was introduced by Hurst and Habetler [68]. In order to locate and extract the RSH, MEH and a recursive algorithm were used to ascertain the parameters R/p and ν.

In FFT techniques, the frequency resolution has a direct relation to signal capture time: the longer the capture time is, the better the resolution. Nevertheless, in the field of electric motor control, the length of data records is limited in order to obtain an appropriate time response. So, since the bases of SSE using FFT were set in [67], the effort was put in both reducing time response without losing accuracy and extending the method to transient conditions.

Considering this, in 1996, a study addressing these problems was presented by Blasco et al [69]. It was stated that even with small data records, it is possible to achieve good accuracy through interpolation, windowing methods and choosing the appropriate RSH from the spectrum. The accuracy was less than 0.1 rpm, and the time response less than 1 s and in occasions much less than 1 s. A transient-state study was also presented. The results showed that the speed predictor followed the real speed after a delay which could be determined. Furthermore, two algorithms to detect RSH, where the number of rotor slots per pole and the parameter ν had to be known in advance, were described. In both methods, the problem about RSH crossing PWM harmonics was addressed. The first one, based on the detection of one RSH, showed reliability from 50 Hz to 5 Hz. The second one, based on the detection of two RSH, improved reliability until 2 Hz. Finally, a discussion about how to generalize the RSH detection for any squirrel cage IM was also presented. It was stated that the main problem was to know *R* and ν as they were parameters that were neither available in the nameplate nor in the motor data-sheet. To work around this issue, they proposed that the motor/drive manufacturer, who can ascertain these parameters, sold each particular motor with its particular RSH detector embedded in the drive or, alternatively, that human operator derived their values through visual inspection of the frequency spectrum.

Since then, many studies have been published aimed at studying and improving different aspects of the technique such as: accuracy, real-time response, applicability, new signal processing methods, computational time and its use in unconventional machines. For example, in 1998 [70], a speed identifier was proposed with an error of less than 0.6 rpm and a real-time speed update of less than a sample period. This was achieved by extracting the RSH via adaptive digital filtering. Years later, in [71,72], the relationship between the number of rotor slots, the number of pole pairs, the stator winding characteristics and the presence of RSH and other harmonics under healthy and eccentricity machine conditions was studied. This led the authors to establish a norm in selecting motors for SSE. In 2006 [4], an optimal slip estimation algorithm embedded in a diagnostic system using MCSA was proposed. This algorithm, assuming that the number of rotor slots was known, used broken bar harmonics, RSH and the Bayesian method of estimation to compute mechanical speed. The accuracy, in terms of slip frequency average error, was 2.97%. In 2013 [73], in order to reduce computation time, a novel approach that did not require spectral analysis was presented. It was based on extracting high order RSH (with previous knowledge of the number of rotor slots) through demodulating the information captured by an external search coil. By this method, a maximum speed error of 0.4% was achieved (≈11 rpm). In 2015 [74], a new RSH-based method to estimate speed in non-stationary conditions was introduced. The proposed algorithm, assuming that the number of rotor slots was known, used short time Chirp-Z transform to search the supply frequency and the RSH. In this case, the maximum deviation found between the estimated and the measured speed using an optical tachometer was: 0.044% (≈0.7 rpm) for the lab test and 0.163% (≈2.4 rpm) for the industrial test. In 2016 [75], in order to improve the dynamics of speed estimation, a new algorithm that combines RSH detection, phase lock loop and AI was presented. In this work, the number of rotor slots was determined by comparing two current sequences at different load conditions with a fixed supply frequency. The maximum speed error in steady-state was found to be 0.083% (≈0.7 rpm). In 2017 [76], MEH was used to estimate the slip in an online and real-time system for detecting partial broken rotor bars. The harmonics were found using a spectral estimator based on Rayleigh quotient theory, achieving a mean square error of 1.91 × 10^−7 ^when compared to a physical sensor. Finally, in recent years, an effort has been made to extend SaEHB techniques to multi-phase IMs [77,78].

### 4.3. Methods Based on Magnetic Anisotropy in Online Diagnosis

SIB methods were born to meet a very specific need for sensorless control: good performance at zero or very low speed. Nevertheless, the most common diagnostic procedures such as MCSA require the motor to be operating with relatively high speed and load. Therefore, these SSE techniques are not suitable to be integrated into this type of automatic fault diagnosis devices, since, outside the low-speed range, the robustness gained does not compensate for their excessive complexity of implementation.

Conversely, SaEHB methods are a great option for diagnostic algorithms via MCSA, since they are:

- Accurate: when based on RSH, errors can be less than 0.1 rpm.
- Robust: they do not depend on any time-varying parameter.
- Easy to implement: no need to subject the motor to a signal other than the one provided by its normal power supply.
- Compatible with MCSA: similar signal processing techniques and speed range.

Despite this, there are some drawbacks that have not been solved yet. On the one hand, in MEH-based methods, only the number of poles need to be known, which is a parameter available on the nameplate. This makes them the preferred methods for commercial diagnostic devices. However, they have a very narrow bandwidth. This means that they only vary a few fractions of hertz over its normal operating range. Therefore, a small error in the estimation of their frequencies implies a large error in the estimation of the speed (see Section 5). On the other hand, RSH-based methods can achieve very accurate estimates due to their wider operating bandwidth. That is the reason why they are so popular in academia, as shown in the literature review of previous subsection. However, these methods need to know the number of rotor slots to work, which is a problem for industry applications, as motor owners are rarely aware of this parameter. Therefore, the applicability of the method is dramatically reduced outside the laboratory.

The estimation of the [R/p,ν] is the main limitation to bring this type of algorithms to industrial scale. However, there is very little research addressing the problem. In fact, most of the papers about RSH-based algorithms either do not provide any information on how to obtain those parameters [4,65,66,73,74,78], or propose non-automatic/invasive methods requiring human visual inspection, which limits their applicability in industrial environments [67,69,70,75]. Only a few papers have proposed self-commission methods to ascertain this set [68,79]. For example, in [68], the method relies on a preliminary speed estimation using the MEH, which, as already stated, is an unreliable source since small errors in frequency estimation mean big errors in speed. Moreover, there is also the disadvantage that MEH often do not manifest themselves with sufficient intensity to be distinguished from the noise level (see Section 5.1.1), being necessary in this case, as the authors comment, a no-load test to determine the number of rotor slots and the index ν associated with each RSH (increasing invasiveness). Something similar happens with the method proposed in [79], where in this case the unreliable source is the preliminary speed estimation based on nameplate data (see Section 5.2.1).

The unreliability of R/p and ν estimation can lead to misdiagnosing the IM due to the reasons exposed in Section 2. For example, Table 1 shows the speed and LSH frequency errors caused by assigning ν=1 and R/p=26 to the actual RSH (−1) for different motors with R/p=28 working at two different slips. As can be seen, the speed error decreases as the number of pole pairs increases or the slip decreases. Yet, looking at the error committed in LSH frequency due to the wrong speed estimation, we find that it is independent of the number of pole pairs and that the error committed. Moreover, this error is of the same magnitude that the one necessary to issue a false positive in Figure 1. Therefore, it is not only necessary for the method to be non-invasive, but also absolutely accurate to avoid false negatives and positives during diagnosis in any type of IM.

Finally, Table 2 summarizes the compatibility analysis between MCSA and the major families of SSE methods. From this analysis, it can be concluded that RSH-based methods are the best option for accurate diagnosis. Nevertheless, there is not yet a method to obtain R/p and v in a reliable, automatic and non-invasive way. That is precisely what makes RSH methods currently unsuitable for its use in high-reliability and high-applicability automatic diagnostic procedures via MCSA, and therefore, an aspect where academia should put its efforts.

## 5. Commercial Devices

In this section, the two industry-leading commercial devices for IM diagnosis are analyzed. The analysis focuses on the speed-dependent online tests performed by these devices to detect motor faults. In this regard, Section 5.1 and Section 5.2 first describe both the principles of the SSE algorithm and the diagnostic method (MCSA-based) of each device, and then analyze their weaknesses relying on the use of theoretical examples and real industrial cases from a database of measurements belonging to 79 different IM (Appendix A: industrial motors data), which were taken using a high-resolution DAQ system (Appendix B: DAQ system) in order to reduce the measurement errors as much as possible, and thus to be able to focus only on the characteristics of each SSE algorithm. Finally, in Section 5.3 the results are discussed to highlight possible lines of improvement.

As explained in the following subsections, both commercial devices estimate the speed to approximately calculate the position in the spectrum of the faulty harmonic frequency, set a search frequency band around it, and finally calculate the maximum amplitude harmonic inside. Therefore, one of the key factors for a correct diagnosis is the search window. Window quality depends directly on the accuracy of the speed estimation. If the estimation is very accurate, the center of any search window will be very close to the fault harmonic frequency, which in turn will allow narrowing the band enough to prevent other significant healthy harmonics from entering it (reducing false positives), while still ensuring that it will contain the fault harmonic frequency (reducing false negatives). Therefore, as analyzed below, since both devices may suffer from wrong speed estimations, their capability to accurately assess the motor condition is affected.

### 5.1. EXPLORER 4000

EXP4000 from Megger is a device whose main purpose is to estimate power quality and efficiency in electrical machines. Yet, as it also allows to evaluate the rotor bars condition through MCSA, it has become a very popular tool when it comes to diagnosing this particular fault in induction motors.

The EXP4000 estimates the rotor speed using an algorithm based on detecting the LMEH in a series of instantaneous signals such as: phase current, current vector, sum of imaginary powers, angle of impedance, etc. [87,88]. It should be noted that Equation (3) predicts the frequency of the LMEH (k=1− sign) only for the spectrum of the stator current. According to [88], for the rest of the magnitudes analyzed by EXP4000, the frequency of the LMEH is given by $f_{LMEH}^{*}=f_{0}\left(1-s\right)/p$. Taking this into account, the bases of the algorithm are:

In the stator current spectrum, the LMEH is located in a position slightly higher than $\left(1-1/p\right)f_{0}$ Hz, which is known if *p* is also known. Therefore, the algorithm sets a window whose lower limit is $\left(1-1/p\right)f_{0}$ Hz, which is the result of making s=0 in (3), and whose upper limit is $\left(1-\left(1-s_{max}\right)/p\right)f_{0}$ Hz, being $s_{max}$ the slip correspondent to the maximum expected load (in [88] it is assumed to be $s_{max}=180/n_{sync}$). Then, the maximum peak in the band is recorded assuming it is the LMEH. A similar process is repeated for each signal considered, but taking into account that, in their spectra, the formula that predicts the LMEH frequency is $f_{LMEH}^{*}=f_{0}\left(1-s\right)/p$. Finally, the low amplitude peaks are discarded and the speed is estimated as the average of the largest group of estimations whose predictions coincide within a margin of 2 rpm.

As for the evaluation of rotor bars condition, the EXP4000 relies on localizing and quantifying the LSH. To do so, using the estimated speed and applying (1), the device calculates an estimated position for the LSH. Then, it sets a frequency window around this position and quantifies the maximum peak assuming it is the LSH. Finally, the EXP4000 applies the following default criterion to output a diagnosis: no damage to rotor bars if its amplitude is below −45 dB, possible damage to one or several bars if it is between −45 dB and −36 dB, and several broken bars if it is above −36 dB.

#### 5.1.1. Mixed Eccentricity Harmonics: Detectability Problems in Two-Pole  Machines

**Figure 3:**
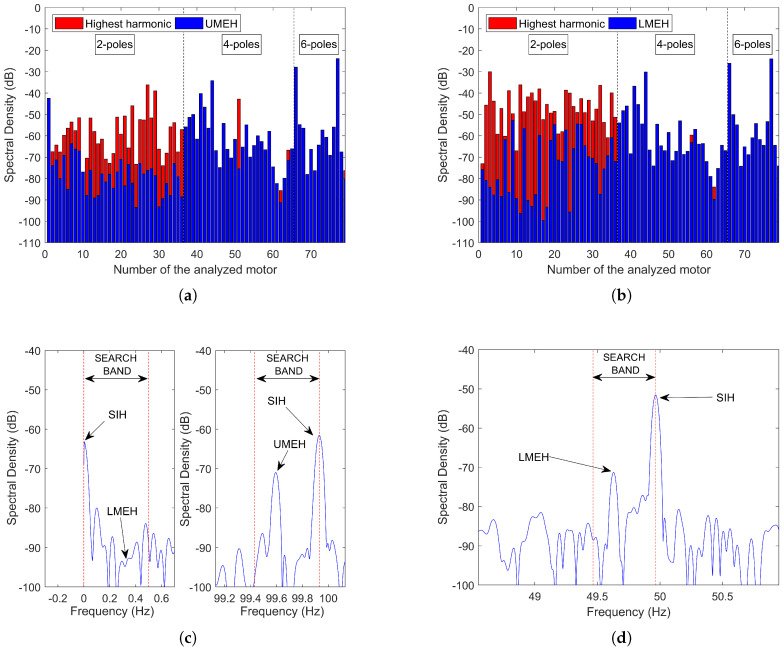
(**a**) UMEH and (**b**) LMEH detectability analysis for 79 IM covering a search band from zero to rated slip in the spectrum of the line current. Example of a two-pole 45 kW IM where: (**c**) the SIH is the highest harmonic in the search band defined for the UMEH and LMEH in the line current spectrum and (**d**) in the search band defined for the LMEH in the instantaneous power signal.

Mixed eccentricity is a problem inherent to any IM. It is caused by inaccuracies in the manufacturing process and misalignments during the motor-load coupling. Therefore, MEH are found in almost any IM, which does not mean that they always have a high amplitude. For example, in new and carefully coupled motors, they are normally only a few dB (if any) above the noise level. Moreover, the presence of nearby harmonics (not associated with motor faults) can complicate the detection process, especially, in two-pole motors (as analyzed below). This particular problem has been studied through the spectral analysis of 79 industrial motors (motor data can be found in Table A1 of Appendix A). The results of the analysis are summarized in Figure 3a (UMEH) and Figure 3b (LMEH), where blue bars represent their amplitudes, and red bars the amplitude of the highest harmonic found in the band determined by (3) when *s* is varied from zero to its rated value. A blue bar completely overlapping the red bar means that the highest harmonic corresponds to the MEH.

The analysis shows that in 48.10% of the cases, there is a higher harmonic than the UMEH in the search band, which for all these cases is the harmonic at $f_{0}+f_{0}/p$ Hz (speed independent and placed at the upper limit of the search band). In two-pole machines, this frequency is $2f_{0}$ Hz (an even multiple of the fundamental), while in four and six-pole machines it coincides with a non-integer multiple of $f_{0}$. Theoretically, there should not be even multiples in the stator line current; however, as no real motor is perfectly symmetric, the existence of this harmonic is very common. Therefore, this explains why MEH detection can be especially difficult in two-pole machines. Figure 3c (right) shows one of these speed-independent harmonics (SIH) belonging to a two-pole 45 kW IM.

Regarding the LMEH, the same detectability problem has been observed with the same proportion of cases (48.10%) where the LMEH is not the highest harmonic in the search band. As with the UMEH, the majority of these motors corresponds to two-pole machines. In this case, the SIH harmonic that is causing problems is placed at 0 Hz (which is the lower limit of the search band), that is, the DC harmonic. Moreover, it has also been observed that, even when this component is filtered, for example, subtracting the mean value of the signal, it is still not possible to locate the LMEH in the line current spectrum, since it is submerged by the noise floor (see Figure 3c (left)). Yet, if other electrical quantities are analyzed, as the EXP4000 does, the LMEH may become visible. For instance, Figure 3d shows the spectrum of the instantaneous power signal of the same two-pole 45 kW IM. In this signal, the LMEH is expected to be at $f_{LMEH}^{*}=f_{0}\left(1-s\right)/p$ Hz. As can be seen, the LMEH is now visible. Nevertheless, the detectability problem does not disappear since there is still an SIH in the upper limit of the band.

#### 5.1.2. Mixed Eccentricity Harmonics: Narrow Bandwidth Implications in Rotor Diagnosis

As mentioned in Section 4.3, MEH cover a narrow frequency bandwidth (from no to full load), which means that small errors in estimating their frequencies (given by (3)), lead to significant errors in the estimated slip. Therefore, as this slip is used to set the search window where to find the fault harmonics, the diagnosis reliability is also affected by errors made in MEH frequency estimation. Next, in order to show more in depth the implications of harmonic bandwidth, we proceed to analyze the error committed in calculating the LSH frequency when slip is estimated from a narrow bandwidth harmonic (MEH) and a wide bandwidth harmonic (RSH).

Combining (1) and (3) with k=1, LSH frequency can be expressed as a function of the MEH and fundamental harmonic frequencies:

(4) $$f_{LSH}=-\left(1\pm 2p\right)f_{0}\pm 2pf_{MEH}$$

An error in the LSH frequency calculation can come either from a wrong frequency estimation of the fundamental harmonic or the MEH. Therefore, if the previous equation is differentiated with respect to these two components and then discretized, we can express the error committed in the LSH frequency as a function of the errors committed in the MEH and fundamental harmonic frequencies:

(5) $$\left\{\begin{array}{c} \frac{df_{LSH}}{df_{0}}=-\left(1\pm 2p\right)\\ \frac{df_{LSH}}{df_{MEH}}=\pm 2p \end{array}\right.\to \Delta f_{LSH}=-\left[1\pm 2p\right]\Delta f_{0}\pm \left[2p\right]\Delta f_{MEH}$$

Finally, following the same reasoning, but in this case combining (1) and (2) with k=1 and $n_{d}=0$, we obtain the error committed in LSH frequency as a function of the RSH and fundamental harmonic frequency errors:

(6) $$\left\{\begin{array}{c} \frac{df_{LSH}}{df_{0}}=-\left(1\pm \frac{2\nu }{R/p}\right)\\ \frac{df_{LSH}}{df_{RSH}}=\frac{2}{R/p} \end{array}\right.\to \Delta f_{LSH}=-\left[1\pm \frac{2\nu }{R/p}\right]\Delta f_{0}+\left[\frac{2}{R/p}\right]\Delta f_{RSH}$$

As the number of rotor bars per poles pairs is normally higher than R/p=14 for the majority of IM, the maximum coefficient multiplying $\Delta f_{RSH}$ is 2/14=0.14, while the coefficient multiplying $\Delta f_{MEH}$ is much higher and increases with the pole pairs: 2p=2,4,6… Assuming ν=+1, which is the most common case, the maximum absolute value of the coefficient multiplying $\Delta f_{0}$ for the RSH is: 1+2/14=1.14, while for the MEH increases as 1+2p=3,5,7… or 1−2p=1,3,5… Therefore, for the same frequency error $\Delta f_{0}$, and the same error estimating the RSH and MEH $\Delta f_{RSH}=\Delta f_{MEH}$, the RSH provides a much more accurate estimation of the LSH frequency. The errors $\Delta f_{0}$, $\Delta f_{RSH}$ and $\Delta f_{MEH}$ can mainly come from three sources:

1. Harmonic misidentification: the harmonic is confused with another close to it.
2. Harmonic in movement during capture time: the harmonic energy spreads over a range of frequencies instead of being concentrated in a single peak.
3. Frequency resolution error: the real harmonic frequency is between two FFT consecutive bins.

Sources 1 and 2 can be neglected for the fundamental harmonic, given that it is the highest harmonic in the spectrum and that its frequency is unlikely to significantly change in a short time capture (5 to 20 s). As for the MEH, source 1 has already been addressed in the previous subsection, while source 2 can be neglected if it is also assumed that speed will not change in a short time period. Therefore, if there is no harmonic misidentification and the regime is stable, the frequency resolution can be considered as the only source of error.

**Figure 4:**
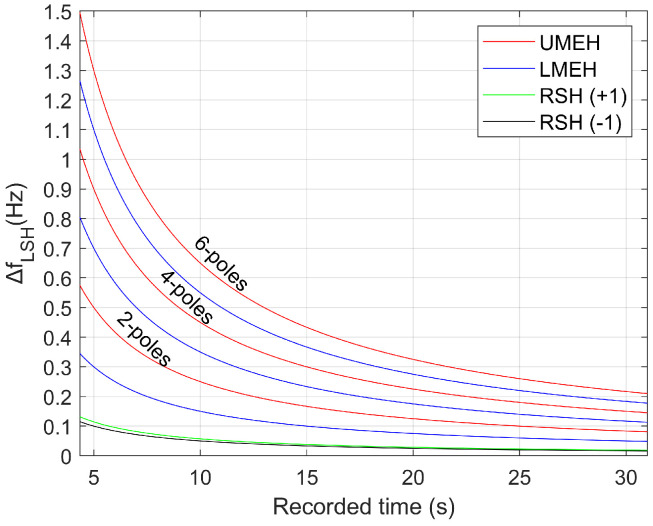
LSH absolute deviation due to the maximum frequency resolution error in RSH, MEH and $f_{0}$.

The FFT is a discrete transform calculated at a set of frequencies starting at 0 Hz, increasing by $1/T_{cap}$ Hz (being $T_{cap}$ the capture time), and ending at half the sampling frequency. Each of these frequencies analyzed is called a bin. Thus, a harmonic whose real frequency is between two consecutive bins is assigned to the closest bin, while spreading part of its energy in the other. This generates the so-called frequency resolution error. In this regard, the maximum frequency resolution error occurs when the actual harmonic frequency is found just in the middle of two bins, which corresponds to $1/(2T_{cap})$ Hz. Figure 4 represents the deviation in LSH frequency (calculated using the absolute value of each term of (5) and (6), so that both errors add up) when the fundamental harmonic, the UMEH, the LMEH and the RSH(±1) frequencies are estimated with an error equal to the maximum frequency resolution error: $\Delta f_{0}=\Delta f_{RSH}=\Delta f_{MEH}=1/\left(2T_{cap}\right)$. This LSH error is quantified for 2p=2, 2p=4, 2p=6 and R/p=28 (typical number for IM). For instance, for an industry-standard capture time of 25 s, which corresponds to a maximum frequency resolution error of 0.02 Hz, the error committed in LSH frequency is, with respect to 0.02 Hz: of the same order for the RSH(±1); 3, 7, or 11 times bigger for the LMEH and 5, 9 or 13 times bigger for the UMEH. It should be noted how inaccurate the estimation of the LSH frequency through MEH could be when compared to a RSH method.

**Figure 5:**
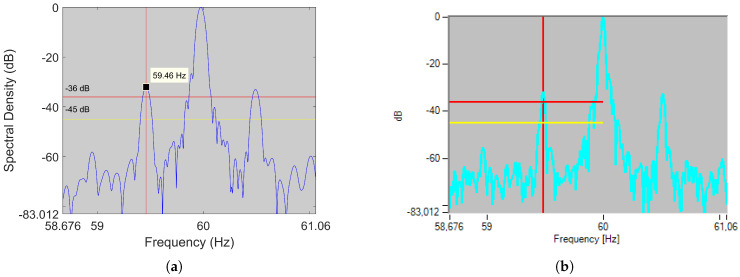
Rotor condition analysis of a four-pole 1500 kW IM using (**a**) a RSH-based algorithm and (**b**) the EXP4000.

**Figure 6:**
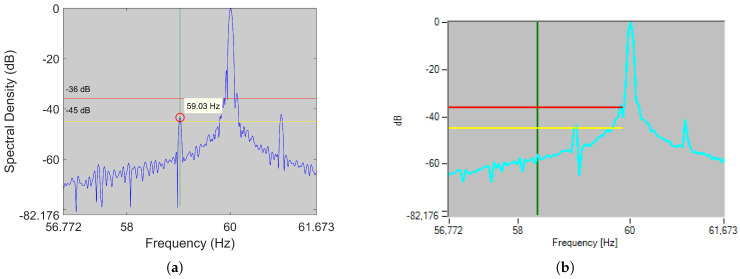
Rotor condition analysis of a six-pole 800 kW IM using (**a**) a RSH-based algorithm and (**b**) the EXP4000.

To show how this error might lead to an erroneous diagnosis, two industrial motors were analyzed using the EXP4000 (based on the LMEH) and a RSH-based algorithm. The first is a four-pole 1500 kW IM, while the second a six-pole 800 kW IM. Table 3 summarizes the results for each machine, while Figure 5 and Figure 6 show the RSH-based algorithm result on the left (applied to the original current captured by the EXP 4000) and the figure generated by the EXP 4000 on the right (in both cases horizontal lines show the default limits for healthy and faulty state used by the EXP4000, while the vertical line shows the frequency at which each algorithm placed the LSH). The differences between the spectra were due to very small differences in signal processing: the way EXP4000 applied the FFT was not perfectly reproduced when analyzing the current extracted from the device, since this information ws not provided by the manufacturer. Nevertheless, since both were practically identical in frequency and amplitude (see the first two rows of Table 3), the differences in diagnosis could be considered to be caused exclusively by the differences between the SSE algorithms.

Regarding the difference in diagnosis, the RSH-based algorithm was able to estimate the LSH frequency very accurately in both motors (Figure 5a and Figure 6a), being the error committed ($f_{LSH,dev}=0.005$ Hz and $f_{LSH,dev}=0.003$ Hz), 7 and 11 times smaller than the frequency resolution ($f_{res}=0.039$ Hz and $f_{res}=0.036$ Hz), thereby issuing a correct diagnosis. Conversely, the EXP4000 was able to diagnose satisfactorily only the first motor (Figure 5b): the error $f_{LSH,dev}=0.039$ Hz was of the same magnitude as the frequency resolution $f_{res}=0.039$ Hz. As for the second motor, it delivered a false negative (Figure 6b): in this case, the deviation with respect to the LSH frequency was $f_{LSH,dev}=0.566$ Hz (16 times bigger than the frequency resolution $f_{res}=0.036$ Hz). This error was larger than the one predicted in (5), which meant that there might be an additional source of error. In this case, it could be a harmonic misidentification, given that the LMEH had an amplitude above the noise level of less than 6 dB in the line current spectrum. Moreover, it should be noted that the 0.566 Hz deviation only corresponded to an error of 0.09 Hz in the MEH frequency estimation, which showed how unreliable a MEH method could be.

Finally, in the signals where the LMEH had a frequency of $f_{LMEH}^{*}=f_{0}\left(1-s\right)/p$ (as the instantaneous power signal), the error in LSH frequency was expressed as:

(7) $$\Delta f_{LSH}=-\left[1\right]\Delta f_{0}+\left[2p\right]\Delta f_{LMEH}^{*}$$

As seen, when compared to (5) and for the same frequency resolution error, the deviation caused by the fundamental frequency was always equal or inferior, while the deviation caused by the LMEH frequency was the same. This improved LSH frequency estimation a bit, since, according to [88], EXP4000 uses several signals where LMEH has a frequency of $f_{0}\left(1-s\right)/p$ Hz. However, the coefficients of (7) were still further from those given by (6).

### 5.2. MCEMAX

MCEMAX is a device from PdMA that conducts three online speed-dependent tests to diagnose rotor asymmetries and eccentricities in an IM: Demodulation Test (DT), Rotor Evaluation Test (RET), and Eccentricity Test (ET). The first is used to estimate the speed, while the others are used to identify, respectively, rotor electrical asymmetries (such as bars breakage) and rotor-stator misalignments.

During the DT, the rotor speed information is extracted from two slip dependent harmonics related to bars breakage and mixed eccentricity [89]. In the spectrum of the demodulated current, they appear respectively, at $f_{BBH}^{Demod}=2sf_{0}$ Hz and $f_{MEH}^{Demod}=f_{0}\left(1-s\right)/p$ Hz. In order to extract their frequencies, a track and find algorithm is performed. Finally, slips and speeds are calculated using the above formulas. The bases of the track and find algorithm are:

A preliminary speed estimation is carried out using nameplate data and current level. Then, with this estimation, $f_{BBH}^{Demod}$ and $f_{MEH}^{Demod}$ are calculated in order to set around each one a frequency band of ±0.3 and ±0.12 Hz, respectively (the rationale for using these fixed limits and not others is not disclosed by the company). Finally, the highest peak within each frequency band is identified. If one peak is below the noise level or speeds do not match, MCEMAX asks the user to estimate speed manually. If both peaks are above noise level and both speeds match, the MCEMAX considers this speed as valid.

Once the speed is estimated, either by the DT or the preliminary estimation, the MCEMAX can assess motor condition via MCSA. Using the RET, it tries to find the LSH in the line current spectrum to assess rotor health. Once this harmonic is identified, if its amplitude exceeds the limit bands set by the manufacturer (first alarm band from −48 dB to −42 dB, second from −42 dB to −36 dB), the device warns the user about a possible damage to rotor bars. Finally, through the ET, the MCEMAX tries to track and find the SEH. In particular, it focuses on the amplitude of those associated with ν=±1 and ν=±3 in (2). In this case, the criterion used is to consider that an eccentricity problem exists if their amplitudes are 20 dB above the noise level.

#### 5.2.1. Implications of Nameplate-Based Approximations

In nameplate-based methods, speed is estimated through linearization of the current-speed curve using two sets of points: $\left[I_{N},n_{N}\right]-\left[I_{0},n_{sync}\right]$. As the no-load current cannot be neglected in IM ($0.9I_{N}>I_{0}>0.20I_{N}$), it has to be estimated, thus being the first source of error. Moreover, for motors with rated power >1 kW, a maximum tolerance of ±20% is allowed on the nameplate rated slip [90,91]. If this is added to the effects of degradation due to use, we have that the rated operation point can be quite far from the one stated on the nameplate. Therefore, making a speed estimation from nameplate values may lead to set frequency bands not containing the harmonics sought, which for the MCEMAX are: the Broken Bars Harmonic (BBH) and the MEH. To study this problem in the 79 industrial motors, the MCEMAX algorithm has been replicated.

Preliminary slip estimation is carried out using the following formula:

(8) $$s_{pre}=\frac{I_{L}}{I_{N}}\cdot s_{N}\cdot k_{exp}$$

where $I_{L}$ and $I_{N}$ are, respectively, the operating and rated current, $s_{N}$ the rated slip and $k_{exp}$ an experimental factor that allows to obtain very similar results to those of the MCEMAX. Then, using this slip, $f_{BBH}^{Demod}$ and $f_{MEH}^{Demod}$ are calculated according to Section 5.2. Finally, a frequency band of ±0.3 Hz for the BBH and ±0.12 Hz for the MEH is set, just as MCEMAX does.

**Figure 7:**
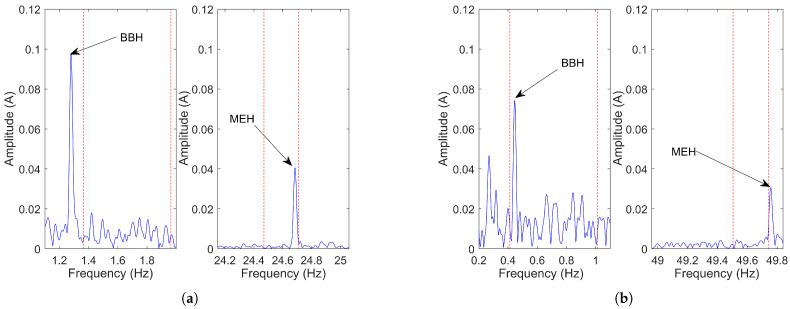
Spectrum of the demodulated current of: (**a**) a four-pole 37 kW IM and (**b**) a two-pole 45 kW IM, with the search windows established by the MCEMAX for the broken bars harmonic (left) and mixed-eccentricity harmonic (right).

Using this algorithm, the actual frequency of the BBH remained outside the search window in 36.71 % of the cases, while the actual frequency of the MEH did so in 32.91% of the cases. Figure 7a shows one of the cases where the BBH remained outside the search band in a four-pole 37 kW IM, with 0.02 rated slip and operating at 87% of the rated current, while Figure 7b shows one of the cases where the MEH is the one that remained outside the search band in a two-pole 45 kW IM, with 0.01 rated slip and operating at 75% of the rated current. Therefore, for the motors analyzed, the device would ask the user for a manual estimation in a considerable number of times, thereby losing its automatic characteristic and leaving the speed estimation quality up to the user’s ability. Finally, if the user tries to visually identify the MEH and BBH, they might not appear as prominent peaks like the ones depicted in Figure 7, and besides, a prominent peak in that area might be caused by another harmonic (as discussed below).

#### 5.2.2. Broken Bars Harmonic: Detectability Problems

The amplitude of the BBH that appears in the spectrum of the demodulated current (used by MCEMAX, together with MEH, to estimate the speed) is related to the amplitude of the harmonics predicted by Equation (1) (with k=1) in the line current spectrum (sideband harmonics). However, this relationship is complex, as it depends on both the constructional parameters of the machine and the characteristics of the load [92].

**Figure 8:**
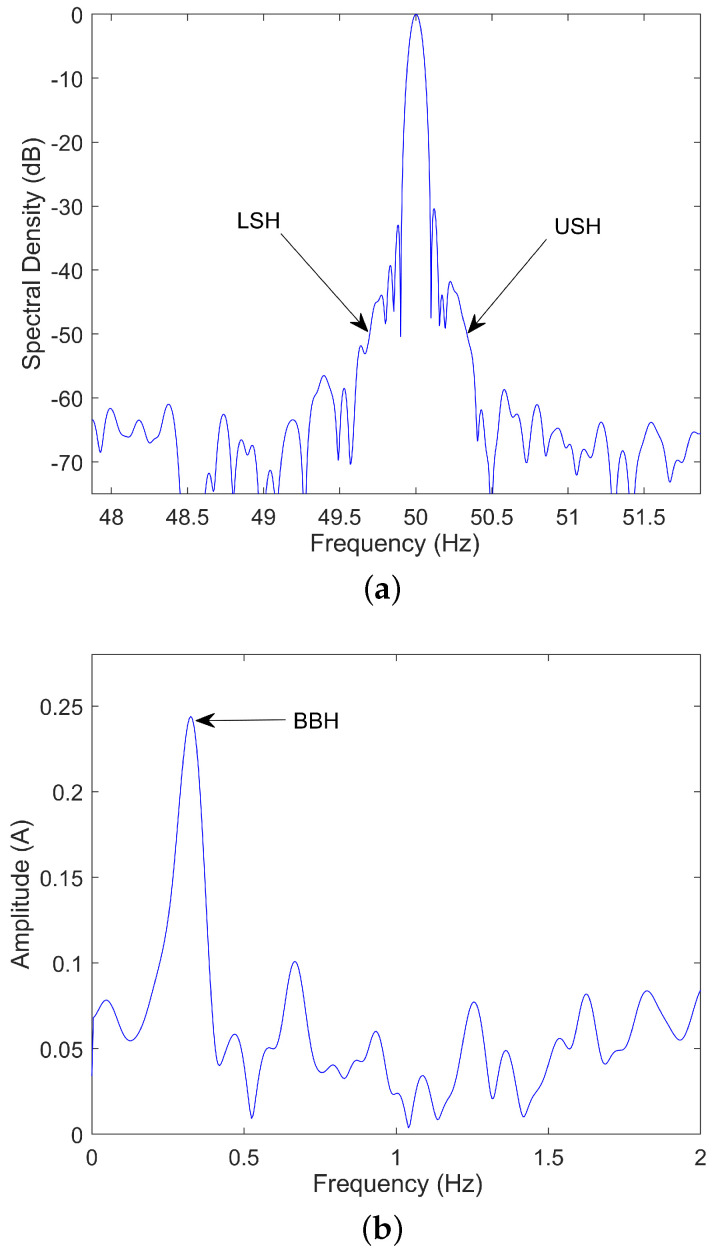
Broken bars harmonics in the spectrum of a two-pole 90 kW induction motor operating at 33.6% of its rated slip: (**a**) stator current and (**b**) demodulated stator current.

In motors that operate with relative high slips, it should be possible to see (if exist) the sideband harmonics in the line current spectrum with a high enough capture time (10 to 40 s). Yet, in motors that operate with very low slip indexes, it may become impossible to detect these harmonics for the same capture time, since they can be masked by the spectral leakage of the fundamental component. Therefore, it is precisely in these cases where the spectrum of the demodulated current offers a great advantage in comparison to the line current spectrum thanks to the removal of this component [93]. In this regard, Figure 8a shows the line current spectrum of a two-pole 90 kW IM operating at 34% of its rated slip, where the sideband harmonics are masked by the spectral leakage of the fundamental component, while Figure 8b shows the spectrum of the demodulated current where the BBH is clearly visible.

Despite the advantage of removing the fundamental component, the detectability of the BBH in the spectrum of the demodulated current can still be problematic, for instance, due to the presence of other nearby harmonics related to load variations or because its amplitude is below the noise floor. In this regard, the effectiveness of estimating speed through this harmonic was tested by analyzing the spectrum of the demodulated current of the 79 industrial motors using two methods. Both of them computed the FFT of the demodulated current, which was calculated as the absolute value of the analytic signal ${\vec{i}}_{h}\left(t\right)=i\left(t\right)+j\cdot H\left(i(t)\right)$, where i(t) is the stator current and *H* the Hilbert transform. Method 1 employed a track and find algorithm consisting of detecting the maximum peak in a frequency band that was calculated as described in the previous subsection, while Method 2 used the same technique but establishing the frequency band around the exact frequency of the harmonic. The aim of Method 2 was to check if, under the most favorable conditions (perfect preliminary speed estimation), there were still problems in detecting the broken bars harmonic.

**Figure 9:**
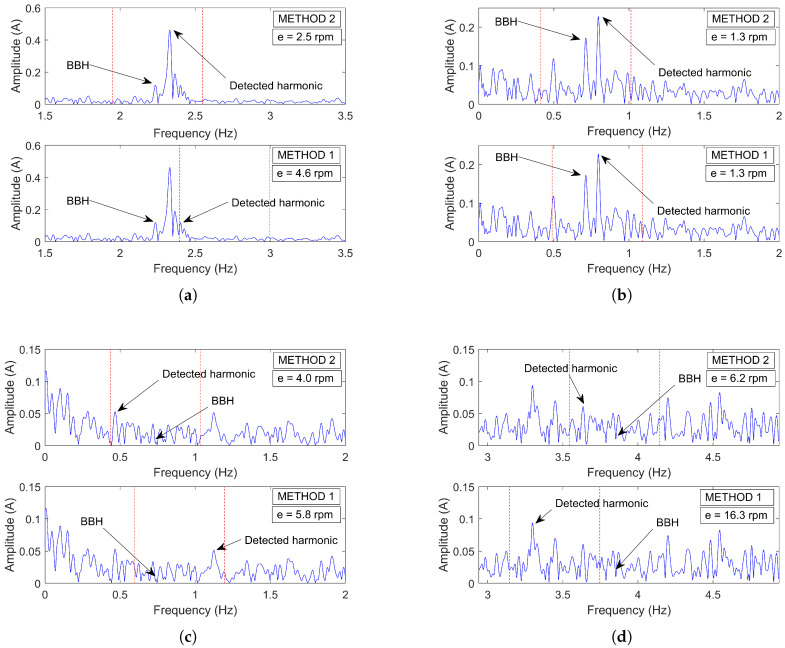
Examples where Method 1 and 2 failed to provide a correct speed estimation in the spectrum of the demodulated current of a: (**a**) two-pole 248 kW IM at 67.7% of its rated slip, (**b**) four-pole 90 kW IM at 60.5% of its rated slip, (**c**) four-pole 55 kW at 61.2% of its rated slip, and (**d**) a two-pole 139 kW IM at 96.1% of its rated slip.

Table 4 shows the number of motors (as a percentage of the total analyzed) whose errors with respect to the actual speed were greater than 0.5, 1.5, 2.5, 3.5 and 4.5 rpm. As can be seen, Method 1 (the one similar to MCEMAX) presented more detectability problems than Method 2. These problems were mostly due to the nameplate-based approximations, as discussed previously. Yet, in the most favorable case (Method 2), where the frequency band covered the broken bars harmonic position, there was also a considerable amount of motors (18.99%) with a speed error greater than 0.5 rpm. In this regard, Figure 9 shows examples of real industrial measures, along with the search bands and absolute speed error, where both Method 1 and 2 failed to provide a correct speed estimation, either for the presence of a higher harmonic in the search band (Figure 9a,b) or an insufficient amplitude of the BBH (Figure 9c,d).

#### 5.2.3. Mixed Eccentricity Harmonic: Detectability and Accuracy Problems

MCEMAX uses both the BBH and the MEH in the demodulated current spectrum to estimate the speed (Section 5.2). As with the BBH, the MEH of the demodulated current spectrum is related to the analogous harmonics in the line current given by Equation (3) with k=1. Unlike what happens with the BBH, the spectrum of the demodulated current does not provide such a relevant advantage at low slips. That is because, in the normal spectrum, the MEH is sufficiently separated from the fundamental component so as not to be masked by its spectral leakage. Thus, the removal of this component does not make a big difference in MEH detection. Yet, it seems logical that the MCEMAX algorithm uses the same spectrum for both MEH and BBH in order to save computational effort.

Since the fundamental component does not influence MEH detectability, the problems may be due to: a search window not covering the actual MEH frequency, the presence of other nearby harmonic with higher amplitude or an MEH amplitude below the noise level. The first problem has already been addressed in Section 5.2.1. As for the second, it was found in Section 5.1.1 that the most problematic harmonics that can cause a misidentification are the ones placed at $f_{0}\left(1\pm 1/p\right)$ Hz in the line current spectrum, particularly, in two-pole machines. In the spectrum of the demodulated current, these harmonics are shifted to $f_{0}/p$, thereby being also close to $f_{MEH}^{Demod}=f_{0}\left(1-s\right)/p$ Hz. However, unlike the EXP4000 algorithm, the MCEMAX uses a frequency band of ±0.12 Hz centered in a preliminary frequency estimation, instead of a search band covering the range from zero to the expected full-load slip. Then, it is less likely that the band will cover the harmonic at $f_{0}/p$. In fact, using the replicated algorithm, it was found that the search band covered this SIH in only 8.86% of the cases and that all belonged to four- and six-pole machines, where the SIH has a very low amplitude. Therefore, detectability problems will be mostly related to an insufficient amplitude of MEH and/or the presence of load oscillations/imbalances harmonics.

Table 5 shows the number of motors (as a percentage of the total analyzed) whose errors with respect to the actual speed were greater than 0.5, 1.5, 2.5, 3.5 and 4.5 rpm. As can be seen, the number of wrong speed estimations increased when compared to BBH (Table 4). The increase was due to both a higher number of motors with an insufficient amplitude of MEH and the fact that this harmonic had a narrower bandwidth. Following a similar reasoning as in Section 5.1.2, the speed errors as a function of the errors committed in estimating MEH or BBH frequencies in the spectrum of the demodulated current were:

(9) $$\left\{\begin{array}{c} \Delta n=\left[\frac{60}{p}\right]\Delta f_{0}-\left[\frac{30}{p}\right]\Delta f_{BBH}^{Demod}\\ \Delta n=\left[0\right]\Delta f_{0}-\left[60\right]\Delta f_{MEH}^{Demod} \end{array}\right.$$

Unlike what happens with MEH, the speed estimation through BBH was not independent of $f_{0}$ error. Yet, as the fundamental frequency tended to be very stable and it was easy to detect, the error committed in estimating it is normally very low when compared to the ones committed with the BBH and the MEH. Therefore, neglecting $\Delta f_{0}$, assuming the same frequency error and for a six-pole machine, the error committed in speed could be six times larger when using the MEH, thereby leading to a mismatch in speed estimations, and therefore, to user intervention, even when both peaks were detected.

#### 5.2.4. Diagnosing with Static Eccentricity Harmonics

Regarding motor eccentricity, the problem relies on using SEH to diagnose (frequencies given by (2) making $n_{d}=0$). These harmonics depend on the number of rotor bars (*R*). Therefore, three scenarios are possible. If *R* and speed are known, which is not usual, MCEMAX automatically locates the SEH and performs the diagnosis as explained in Section 5.2. If *R* is known but not speed, MCEMAX asks the user to manually choose the highest peak in the spectrum to the left of $\left(R/p-1\right)f_{0}$ Hz as the SEH(−1). Then, MCEMAX automatically locates the rest of SEH and estimates the speed from this set. Finally, if *R* is not known, the MCEMAX has a feature to estimate this parameter as long as the speed has been previously estimated either by DT or other means. This feature consists of a manual assistant where the user has to choose a candidate to be the SEH(−1). Next, alleged peaks SEH(+1) and SEH(±3) are automatically detected. Then, the user has to check if the amplitudes and layout of the set is similar to the typical adopted by SEH to finally estimate the number of rotor bars.

**Figure 10:**
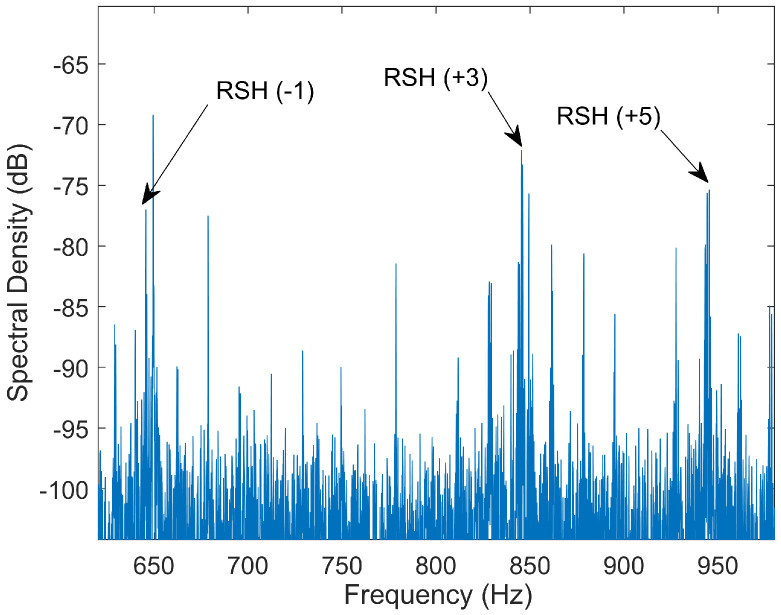
RSH layout in the stator current spectrum of a six-pole 132 kW IM.

This approach has several problems: it is not automatic, requires an advanced knowledge of SEH distribution, and it may easily lead to wrong estimations of *R*. To illustrate the latter, Figure 10 shows the spectrum of a six-pole 132 kW IM. In it, instead of a set of ±1 and ±3 harmonics where the −1 is the one of highest amplitude (which is a common but not always true assumption when identifying RSH or SEH), what we have is a set of −1, +3 and +5 where +3 is the one of highest amplitude. Hence, the user could identify the +3 as the −1, and therefore, estimate the number of bars as 54 instead of the actual 42 bars. That is the reason why the manufacturer asks the owner to verify this number through the motor provider or motor workshop. Yet, as this is not always possible, the applicability of the test is reduced.

### 5.3. Discussion and Lines of Improvement

Regarding MCEMAX, two main drawbacks has been identified in their SSE algorithm. The first, found in the initial stage of its SSE algorithm, is the error made in estimating the center of the band in which the MEH and BBH are sought. This error is mainly due to the no-load current and the fact of assuming as valid the nameplate rated speed (where the norm allows a tolerance in slip up to 20%). This, along with the bands used for each harmonic, causes that, for the 79 industrial motors analyzed, the BBH is outside the search window in 36.71% of the cases, while the MEH in 32.91%. The second drawback, related to detectability and accuracy, is found in the last stage of the algorithm. On the one hand, it has been proved that, even in the case of the band covering the BBH or MEH frequency, their detection can still be problematic, since they can be submerged by the noise floor or be confused with other healthy state harmonics caused, for example, by load oscillations. In particular, the speed error has been higher than 0.5 rpm in 18.99% of the cases for the BBH, and in 29.11% of the cases for the MEH. On the other hand, it has also been analyzed theoretically the disparity between the errors in speed estimation when using the BBH and the MEH of the demodulated current. The analysis has shown that the MEH speed error can be from two times (two-pole machine) to eight times (eight-pole machine) larger than the BBH for the same resolution error, which means that, even if both peaks are detected, the MCEMAX could ask for user intervention.

The first problem can be mitigated without changing the basis of the algorithm by making the search bands of each harmonic proportional to their bandwidths. MEH and BBH bandwidth are, respectively, $sf_{0}/p$ and $2sf_{0}$, that is, the BBH bandwidth is 2p times bigger than the MEH one. Then, if we assumed a fixed search band for the MEH of ±0.12 Hz, the BBH window should have an amplitude of ±0.12·p Hz. Applying this change to the replicated MCEMAX algorithm, the percentage of cases where the BBH remains outside the search window is reduced from 36.71% to 32.91%, therefore, being equal to that of the MEH.

As for the EXP4000, two main drawbacks has also been identified. The first is related to LMEH detectability in the search band covering from zero to the full-load expected slip. It has been shown in Section 5.1.1 that there is a high-amplitude SIH in two-pole machine placed at 0 Hz (line current) or $f_{0}$ Hz (current vector, instantaneous power…) that reduces LMEH detection. Two possible solutions can be applied to avoid this harmonic: to displace the band or to filter the SIH. The first solution requires a good enough frequency resolution so as not to lose too much detectability range. For example, a two-pole high-power IM, where rated slip can be as low as 0.5%, fed at 40 Hz gives us a MEH frequency bandwidth of 0.2 Hz. This means that a search band displacement of only 0.1 Hz (the frequency resolution of a 10 s record) would translate into losing 50% of the detectability range. The second solution, requires the filter to be as sharp as possible for the same reasons, thereby complicating the algorithm. Yet, even in the case of this SIH being filtered or not included in the search band, there might still be detection problems caused by an insufficient amplitude of the LMEH or the presence of nearby harmonics as in the case of MCEMAX. The second drawback is related to LMEH accuracy. As discussed in Section 5.1.2, small errors in estimating LMEH lead to large errors in LSH frequency, and therefore, to the possibility of issuing wrong diagnosis.

As discussed along the section, both devices suffer from detectability and accuracy issues due to the harmonics they use. This might be improved if RSH were used instead, since they appear at higher frequencies (far from other high-amplitude harmonics) and since they have a wider bandwidth (better accuracy). In this regard, three RSH-based algorithms has been tested with the data of the 79 industrial motors so as to compare their performance with the MCEMAX and EXP4000 alike algorithms. These algorithms are:

1- RSH-based algorithm using a search band covering from s=0 to $s=s_{N}$.
2- RSH-based algorithm equal to 1 but displacing the band 0.5 Hz so as not to cover the harmonic at ($n\cdot f_{0}$).
3- RSH-based algorithm using a search band centered in a preliminary frequency estimation using nameplate data. The band amplitude is proportional to that of the MCEMAX for the MEH (±0.12 Hz).
4- The replicated MCEMAX algorithm. A speed match is considered if the difference between BBH and MEH speeds are less than 0.5 rpm.
5- LMEH-based algorithm using a search band covering from s=0 to $s=s_{N}$. Speed is obtained as the weighted average of the ones extracted from the spectrum of the line current and the absolute value of the current vector (similar to EXP4000).

Table 6 summarizes the results of this analysis. As can be seen, the RSH-based algorithms (Alg. 1, Alg. 2 and Alg. 3), where the number of rotor bars is already known, outperforms the MCEMAX-like (Alg. 4) and EXP4000-like (Alg. 5). Despite the clear advantage of using RSH algorithms for SSE, the current lack of a method to obtain the number of rotor bars in an automatic, online, and reliable way prevents its use in industrial devices such as MCEMAX or EXP4000.

Finally, it is fair to mention that, despite the weaknesses highlighted in this section, MCEMAX and EXP4000 are still powerful and user-friendly tools that can be useful in certain conditions to assess rotor bars or eccentricity, as demonstrated by their wide use in industry.

## 6. Conclusions

In the field of induction motor diagnosis via MCSA, even if some papers have mentioned the necessity of accurate SSE, this is the first to highlight and analyze its importance, especially for the modern industry. The analysis presented in this paper is meant to achieve a large consensus on how important it is to have accurate speed information in order to reduce both false positives and negatives. Moreover, to date, there is no research that has gone into an in-depth assessment of the problems and challenges behind obtaining a method to achieve an accurate and automatic speed estimation that is valid for any motor. This is a key step towards Industry 4.0. In this regard, the paper fills this gap for the first time by investigating the major families of SSE techniques, which were mainly conceived for sensorless control, from the perspective of its application in the diagnosis of the induction motor, showing the lacks that yet remain unsolved. In addition, the investigation is enhanced with the analysis, supported by a database of measurements belonging to 79 different induction motors, of the SSE algorithms of the two leading commercial devices highlighting their weaknesses and lines of improvements. From those analyses, it is concluded that:

- FMB methods are apparently not the most suitable for portable devices, since they are invasive due to the need of voltage sensing and a first stage of parameter identification. However, they can be a good option for a continuous monitoring system, since, if implemented in the driver, the set formed by the MCSA diagnostic procedure and FMB method could use the same voltages and current measurements than the control system, as well as take advantage of the natural stops for the parameter identification. Nevertheless, further research is still necessary in order to study whether the accuracy provided by a method like this is enough for its use in high-reliability diagnostic procedures via MCSA.
- SIB methods introduce excessive complexity either for a portable device or for a continuous monitoring system. The complexity is only compensated by their performance at low or zero speed, which is not the range where MCSA is used. Thus, it can be discarded as a promising candidate for SSE in online diagnosis via MCSA.
- SaEHB are the best option for continuous or occasional monitoring due to its compatibility with MCSA in terms of accuracy, robustness, ease of implementation and independence to parameter variations. Among them, MEH-based methods are preferred in industry since they only depend on the number of pole pairs, which is a parameter available on the nameplate. As a counterpart, they provide low accuracy due to the narrow bandwidth of these harmonics. Conversely, RSH-based methods are preferred in academy since they provide higher accuracy, being the drawback in this case its reduced applicability due to the need of knowing the number of rotor slots, which is a parameter rarely known by motor owners.
- Current SaEHB techniques used in commercial devices do not provide reliability in a considerable amount of cases. On the one hand, EXP4000 has the main problem of using MEH, which do not provide enough accuracy due to its narrow bandwidth and the difficulty of being detected, particularly in two-pole machines. On the other hand, MCEMAX uses the BBH and MEH of the demodulated current. In this case, the major drawback is that to locate these harmonics, it uses an algorithm that depends on a preliminary speed estimation whose accuracy is subjected to the magnitude of the no-load current and the consistency between nameplate data and actual values. Nevertheless, detection difficulties also arise in these two harmonics, and besides, their different speed estimation errors may generate inconsistencies, leading the device to ask for a human check.

Thus, commercial diagnostic systems still need a reliable SSE algorithm. According to scientific literature, RSH-based methods are the ones that can provide the highest accuracy and reliability due to its wider bandwidth and ease of detection. Nevertheless, they lack of a general and non-invasive method to automatically determine the number of rotor slots (*R*) and the order of the time harmonic associated to each RSH (ν). This prevents them from being efficiently integrated into Industry 4.0 smart diagnostic systems. Therefore, the authors conclude that, despite SSE techniques have been investigated for a long time in the field of controlled AC drives, the scientific community has yet to provide a precise, automatic and general method that helps to achieve a highly reliable MCSA-based online condition monitoring system for the modern industry.

## Figures and Tables

**Table 1:** Speed and LSH frequency errors caused by assigning ν=1 and R/p=26 to the actual RSH(−1) for different motors with R/p=28 working at two different slips.

	p = 1, s = 0.01	p = 1, s = 0.03	p = 2, s = 0.01	p = 2, s = 0.03	p = 3, s = 0.01	p = 3, s = 0.03
Speed error (rpm)	2.31	6.92	1.15	3.46	0.76	2.30
LSH error (Hz)	0.08	0.23	0.08	0.23	0.08	0.23

**Table 2:** Compatibility analysis between MCSA and the major families of SSE methods.

	Methods	MRAS	EKF	AI	SIB	RSH-Based	MEH-Based
Characteristics							
Only one current sensing		x	x	x	x	🗸	🗸
No need for voltage sensing		x	x	x	x	🗸	🗸
No need for an additional power supply		🗸	🗸	🗸	x	🗸	🗸
Highly accurate estimations (<1 rpm)		x	x	x	x	🗸	x
High speed as one of the target zones of operation		🗸	🗸	🗸	x	🗸	🗸
Insensitive to parameter variations		x	x	🗸	🗸	🗸	🗸
No need for previous training		🗸	🗸	x	🗸	🗸	🗸
No need for parameter estimation		x	x	🗸	x	x	🗸
Simple implementation		🗸	🗸	🗸	x	🗸	🗸
Compatibility with MCSA		Medium	Medium	Medium	Low	High	High

**Table 3:** Results of the rotor condition analysis for a four-pole 1500 kW IM and for a six-pole 800 kW IM. Frequencies are in Hz, speeds in rpm and amplitudes in dB.

	$f_{\mathrm{res}}$	$f_{0,\mathrm{est}}$	$n_{\mathrm{est}}$	$s_{\mathrm{est}}$	$f_{\mathrm{LSH},\mathrm{est}}$	$f_{\mathrm{LSH},\mathrm{real}}$	$f_{\mathrm{LSH},\mathrm{dev}}$	Amp.	Diag.
EXP4000	0.039	59.98	1792.2	0.0040	59.501	59.462	0.039	−31.84	Damaged
RSH-based algorithm	0.039	59.98	1791.6	0.0042	59.467	59.462	0.005	−31.99	Damaged
EXP4000	0.036	60.01	1184.7	0.0129	58.463	59.029	0.566	−56.04	Healthy
RSH-based algorithm	0.036	60.01	1190.4	0.0081	59.032	59.029	0.003	−43.46	Damaged

**Table 4:** Number of motors, as a percentage of the total analyzed, whose errors with respect to the actual speed are greater than 0.5, 1.5, 2.5, 3.5 and 4.5 rpm, when BBH is used to estimate speed.

	>0.5 rpm	>1.5 rpm	>2.5 rpm	>3.5 rpm	>4.5 rpm
Method 1	45.57%	43.04%	37.97%	35.44%	27.85%
Method 2	18.99%	12.66%	7.59%	6.33%	2.53%

**Table 5:** Number of motors, as a percentage of the total analyzed, whose errors with respect to the actual speed are greater than 0.5, 1.5, 2.5, 3.5 and 4.5 rpm, when MEH is used to estimate speed.

	>0.5 rpm	>1.5 rpm	>2.5 rpm	>3.5 rpm	>4.5 rpm
Method 1	50.63%	44.30%	41.77%	40.51%	39.24%
Method 2	29.11%	25.32%	24.05%	22.78%	17.72%

**Table 6:** Number of motors, as a percentage of the total analyzed, whose errors with respect to the actual speed are greater than 0.5 rpm for 5 different algorithms.

	Alg. 1	Alg. 2	Alg. 3	Alg. 4	Alg. 5
Error > 0.5 rpm	21.52%	5.06%	36.71%	65.82%	51.90%

## Data Availability

Not applicable.

## Abbreviations and Formula Nomenclature

The following abbreviations and formula nomenclature are used in this manuscript:

SSE	Sensorless Speed Estimation
MCSA	Motor Current Signature Analysis
IM	Induction Motor/s
FFT	Fast Fourier Transform
FMB	Fundamental Model-Based
MRAS	Model Reference Adaptive System/s
EKF	Extended Kalman Filter
AI	Artificial Intelligence
ANN	Artificial Neural Network/s
FL	Fuzzy Logic
GA	Genetic Algorithm/s
MAB	Magnetic Anisotropy-Based
SIB	Signal Injection-Based
SaEHB	Slotting and Eccentricity Harmonics-Based
USH	Upper Sideband Harmonic
LSH	Lower Sideband Harmonic
RSH	Rotor Slot Harmonic/s
SEH	Static Eccentricity Harmonic/s
DEH	Dynamic Eccentricity Harmonic/s
MEH	Mixed Eccentricity Harmonic/s
LMEH	Lower Mixed Eccentricity Harmonic
UMEH	Upper Mixed Eccentricity Harmonic
BBH	Broken Bars Harmonic
SIH	Speed Independent Harmonic
DT	Demodulation Test
RET	Rotor Evaluation Test
ET	Eccentricity Test
*n*	Speed
$n_{sync}$	Synchronous speed
$n_{N}$	Nominal speed
$f_{MEH}$	Mixed Eccentricity Harmonic/s frequency in the spectrum of the stator current
$f_{LMEH}^{*}$	Lower Mixed Eccentricity Harmonic frequency in the spectrum of quantities other than stator current (EXP4000)
$f_{MEH}^{Demod}$	Mixed Eccentricity Harmonic frequency in the spectrum of the demodulated stator current (MCEMAX)
$f_{BBH}$	Broken Bar Harmonics frequency in the spectrum of the stator current
$f_{BBH}^{Demod}$	Broken Bar Harmonic frequency in the spectrum of the demodulated stator current (MCEMAX)
$f_{LSH}$	Lower Sideband Harmonic frequency in the spectrum of the stator current
$f_{RSH}$	Rotor Slot Harmonic/s frequency in the spectrum of the stator current
$f_{res}$	Frequency resolution
$I_{N}$	Nominal current
$I_{0}$	No-load current
$I_{L}$	Operating current
$k_{exp}$	Experimental factor to correct slip estimation in MCEMAX alike algorithm
$f_{h}$	Generic harmonic frequency
$f_{0}$	Fundamental component frequency
*p*	Number of pole pairs
*s*	Slip
*R*	Number of rotor bars
*k*	Positive integer number (1,2,3…)
$n_{d}$	Positive integer number (0,1,2…)
ν	Order of the stator time harmonic present in the power supply (1,3,5…)

## Appendix A. Industrial Motors Data

This database is the result of several years of consulting work with different types of industry. As a consequence, there are signals of motors driving vertical/horizontal pumps, conveyors, compressors, worm gears, shredders, etc. This fact, together with the wide range of powers and slips, makes the database a robust statistical sample to evaluate the bases of the algorithms described in this paper.

**Table A1:** Industrial motors data.

2-Poles				4-Poles				6-Poles			
$P_{N}\left(\mathrm{kW}\right)$	$s_{N}$	$s/s_{N}\left(\%\right)$	Fig.	$P_{N}\left(\mathrm{kW}\right)$	$s_{N}$	$s/s_{N}\left(\%\right)$	Fig.	$P_{N}\left(\mathrm{kW}\right)$	$s_{N}$	$s/s_{N}\left(\%\right)$	Fig.
1.50	0.047	32.27%		0.75	0.070	3.23%		132.00	0.011	52.45%	Figure 10
30.00	0.017	71.34%		0.75	0.070	9.39%		132.00	0.011	49.51%	
30.00	0.017	69.81%		0.80	0.040	74.05%		132.00	0.011	52.95%	
30.00	0.033	141.56%		0.80	0.040	96.82%		200.00	0.010	47.23%	
45.00	0.010	43.23%	Figure 3 and Figure 7b	1.10	0.057	100.44%		250.00	0.030	16.41%	
55.00	0.013	62.78%		1.10	0.057	10.21%		250.00	0.010	39.05%	
63.00	0.033	50.22%		2.20	0.053	79.05%		253.00	0.010	65.79%	
90.00	0.010	33.63%	Figure 1 and Figure 8	2.98	0.070	24.83%		253.00	0.010	93.75%	
90.00	0.033	66.56%		37.00	0.020	53.90%	Figure 7a	253.00	0.010	65.96%	
110.00	0.007	56.17%		37.00	0.020	44.62%		253.00	0.010	59.46%	
112.00	0.033	75.18%		45.00	0.014	36.01%		375.00	0.014	65.72%	
112.00	0.033	69.43%		55.00	0.012	61.22%	Figure 9c	375.00	0.014	37.98%	
112.00	0.033	88.00%		75.00	0.010	62.68%		375.00	0.014	33.60%	
112.00	0.033	98.72%		90.00	0.013	24.93%		800.00	0.017	48.82%	Figure 6
112.00	0.033	81.50%		90.00	0.015	60.51%	Figure 9b				
117.00	0.033	69.97%		110.00	0.013	30.25%					
132.00	0.007	76.98%		110.00	0.008	73.78%					
132.00	0.007	99.54%		110.00	0.008	83.85%					
134.00	0.033	62.57%		110.00	0.008	72.16%					
134.00	0.033	81.81%		110.00	0.008	100.47%					
139.00	0.040	96.08%	Figure 9d	160.00	0.013	36.90%					
150.00	0.033	79.94%		160.00	0.007	84.36%					
171.00	0.017	90.23%		160.00	0.007	91.88%					
223.00	0.033	74.69%		160.00	0.007	61.25%					
230.00	0.040	86.26%		160.00	0.007	68.10%					
231.00	0.033	68.59%		160.00	0.007	39.65%					
232.00	0.033	74.96%		300.00	0.010	58.53%					
234.00	0.033	88.36%		580.00	0.008	71.90%					
239.00	0.028	81.77%		1500.00	0.007	58.57%	Figure 5				
248.00	0.033	67.73%	Figure 9a								
248.00	0.033	61.55%									
248.00	0.033	65.63%									
270.00	0.033	96.27%									
270.00	0.033	61.85%									
372.00	0.033	44.23%									
2200.00	0.012	36.01%									

## Appendix B. DAQ System

The measurements of the 79 industrial motors were performed with the following DAQ system:

- High-resolution oscilloscopes:
  – PicoScope 4262:
    * Channels: 2.
    * Vertical resolution: 16-bit.
    * Sampling frequency: 10 MS/s.
  – PicoScope 4824:
    * Channels: 8.
    * Vertical resolution: 12-bit.
    * Sampling frequency: 80 MS/s.
- High-precision current probes:
  – TA189:
    * Measuring range: 30 A.
    * Accuracy: 1% of reading ±2 mA.
    * Frequency range: DC to 100 kHz.
  – TA167:
    * Measuring range: 200/2000 A.
    * Accuracy (0–200/1500 A): 1% of reading ±100/± 500 mA.
    * Accuracy (1500–2000 A): ±5 % of reading.
    * Frequency range: DC to 20 kHz.

Regarding the characteristics of the signals, they were always recorded with a minimum capture time of 100 s, at 10 kHz and adjusted scale. Finally, for the analyses performed on the 79 induction motors in this paper, the signal duration was shorten to 81.9 s, as this is the maximum recording time allowed by MCEMAX.
